# Evaluation of Risk Factors for Exacerbations in Children with Adenoviral Pneumonia

**DOI:** 10.1155/2020/4878635

**Published:** 2020-07-31

**Authors:** Na Xu, Peng Chen, Ying Wang

**Affiliations:** Department of Respiratory Medicine, Wuhan Children's Hospital, Tongji Medical College, Huazhong University of Science and Technology, Wuhan, China

## Abstract

**Purpose:**

The aim of this work was to analyze clinical features and laboratory findings of children with adenovirus pneumonia and guide clinical diagnosis, treatment, and assessment of disease severity. *Material and Methods*. Retrospective analysis of clinical data of 285 children with adenoviral pneumonia who were hospitalized in Wuhan Children's Hospital from December 2018 to October 2019. According to the assessment criteria for severe pneumonia, it was divided into the severe group (92 cases) and the nonsevere group (193 cases). Collected clinical manifestations, complications, and laboratory test indicators in two groups of children and conducted all statistical analyses.

**Results:**

The risk of fever and wheezing was significantly higher in the severe group than in the nonsevere group. The difference was statistically significant (*P* < 0.05). The risk of complications in the severe group was significantly higher than that in the nonsevere group. The difference was statistically significant (*P* < 0.05). The levels of AST, LDH-L, PCT, ferritin, and D-dimer in the severe group were significantly higher than those in the nonsevere group. The difference was statistically significant (*P* < 0.05).

**Conclusion:**

Children with severe adenovirus pneumonia have severe clinical manifestations and many complications. AST, LDH-L, PCT, ferritin, and D-dimer levels have important clinical implications for assessing disease severity.

## 1. Introduction

Adenoviruses (AdV) are DNA viruses that typically cause infections involving the upper or lower respiratory tract, gastrointestinal (GI) tract, or conjunctiva. Adenovirus infections are more common in young children, which is more common in 6-month to 2-year-old infants owing to lack of humoral immunity [1, 2]. Fatality rates for untreated severe AdV pneumonia or disseminated disease may exceed 50%. Severe adenoviral pneumonia has an acute onset, severe illness, rapid progress, and a poor prognosis [3]. Compared with previous years, the incidence of severe adenoviral pneumonia in the winter and spring seasons of 2018 in Hubei has increased. This study analyzed clinical data of 285 children with adenoviral pneumonia admitted in our hospital and provided evidence-based medical evidence for the early diagnosis, timely and active treatment of severe cases, and improved prognosis.

## 2. Material and Methods

### 2.1. Patients and Specimen Collection

A retrospective study was conducted on children hospitalized in the Department of Respiratory Medicine, Wuhan Children's Hospital, Tongji Medical College, Huazhong University of Science and Technology, from December 1, 2018, to October 31, 2019. During the study period, it was recommended that all children admitted to the hospital undergo routine testing for respiratory viruses, bacteria, and fungi. 285 cases of adenoviral pneumonia treated in our hospital were selected as the research objects.

### 2.2. Assessment Criteria

According to the symptoms and signs presented in the children, 285 children with adenoviral pneumonia were divided into the severe group (92 cases) and the nonsevere group (193 cases). The severe group meets the assessment criteria for severe pneumonia in children proposed by the British Thoracic Society (BTS) [4]: (i) body temperature > 38.5°C, severe symptoms of systemic poisoning, or hyperthermia; (ii) severe difficulty in breathing, obvious cyanosis, lung rale or signs of pulmonary consolidation, or chest X-rays showing flaky shadow; (iii) heart failure, respiratory failure, toxic encephalopathy, microcirculation disorders, shock, or any of them; (iv) combined empyema, pneumothorax, and (or) those with sepsis and toxic intestinal paralysis; and (v) those with multiple organ dysfunction. Among them, (i) and (ii) are indispensable conditions, and any one of (iii), (iv), and (v) can be diagnosed as severe pneumonia.

### 2.3. Specimen Testing

All the nasopharyngeal swab specimens of the 285 children were within 24 hours of admission and tested for influenza virus, PIV, respiratory syncytial virus, Epstein-Barr virus, Cytomegalovirus, coxsackievirus, and AdV by use of a direct immunofluorescence test. Simultaneously, the collected sputum samples were tested for bacterial and fungal culture, and venous blood was drawn to complete blood routines, C-reactive protein (CRP), procalcitonin (PCT), serum amyloid A (SAA), erythrocyte sedimentation, ferritin, liver function, myocardial enzyme spectrum, coagulation, and other tests. Bronchoalveolar lavage was performed in 76 children of the severe group. The bronchoalveolar lavage fluid was sent to BGI Genomics for pathogenic microorganism high-throughput sequencing detection.

### 2.4. Statistical Analysis

The qualitative variables were compared by a Chi-squared test or Fisher's exact test, as appropriate, and the quantitative variables by Student's *t*-test. And *P* < 0.05 was considered statistically significant. All statistical analyses were performed using SPSS 21.0.

## 3. Results

### 3.1. General Situation

Among the 285 cases, 180 were males and 105 were females, and the male to female ratio was 1.7 : 1. The mean age of the 285 patients was 1.7 years, ranging from 1 year to 5 years. According to the assessment criteria of the disease, they were divided into the severe group (92 cases) and the nonsevere group (193 cases).

### 3.2. Clinical Manifestations and Physical Sign

Common clinical manifestations and physical signs of adenoviral pneumonia are fever, cough, wheezing, lung rales, etc. There was no significant difference in the incidence of cough and lung rales between the severe and nonsevere groups (*P* > 0.05). The incidence of fever and wheezing in the severe group was significantly higher than that in the nonsevere group, and the difference was statistically significant (*P* < 0.05) (see Table 1).

### 3.3. Complications

Complications of adenoviral pneumonia are divided into intrapulmonary complications and extrapulmonary complications. Intrapulmonary complications were mainly respiratory failure, pleural effusion, massive consolidation, and atelectasis; long-term complications such as bronchiolitis obliterans and bronchiectasis are not included. Except for diarrhea, intrapulmonary complications in the severe group were significantly higher than those in the nonsevere group, and the difference was statistically significant (*P* < 0.05). Extrapulmonary complications are mainly liver injury, myocardial damage, electrolyte disturbances, diarrhea, coagulation disorders, and toxic encephalopathy. Liver injury, coagulation disorders, and toxic encephalopathy were significantly higher in the severe group than in the nonsevere group, and the difference was statistically significant (*P* < 0.05) (see Table 2).

### 3.4. Comparison of Peripheral Blood Test Results

The tests contain peripheral blood leukocyte counts, C-reactive protein (CRP), procalcitonin (PCT), serum amyloid A (SAA), erythrocyte sedimentation rate (ESR), ferritin, aspartate aminotransferase (AST), lactate dehydrogenation enzyme (LDH-L), and D-dimer. The levels of PCT, ferritin, AST, LDH-L, and D-dimer in the severe group were significantly higher than those in the nonserious group (*P* < 0.05). The white blood cell count, CRP, SAA, and ESR were not significantly different between the two groups (*P* > 0.05) (see Table 3).

### 3.5. Characteristic of Pathogens in Patients with Mixed Infection

In the severe group, 61 cases (*n* = 92, 66.30%) had mixed infections with other pathogens, including 34 cases of mycoplasma, 23 cases of bacteria, 6 cases of fungi, 10 cases of other viruses, and 15 cases with multiple pathogens (≥3, including adenovirus). In the nonsevere group, 84 cases (*n* = 193, 43.52%) had mixed infections, including 54 cases of mycoplasma, 23 cases of bacteria, 0 cases of fungi, 21 cases of other viruses, and 19 cases with multiple pathogens. Among bacterial infections, H. influenzae is more common, with 17/23 and 17/23 in the two groups. Six cases of fungal infections in the severe group were Aspergillus infections. Among patients infected with other viruses, 5/10 cases in the severe group were EB virus, 2/10 were Cytomegalovirus, 2/10 were respiratory syncytial virus, and 1/10 were parainfluenza virus; in the nonsevere group, 12/21 is EB virus and 9/21 is Cytomegalovirus. Comparing the data of mixed infections between the two groups, we found that the mixed infection rate of the severe group was significantly higher than that of the nonsevere group (66.30% vs. 43.52%).

## 4. Discussion

Adenovirus is a nonenveloped double-stranded DNA virus that exists widely in nature. The detection rate of adenovirus pneumonia varies in different countries and regions. The incidence of adenovirus pneumonia in China is high, and it occurs frequently in spring and winter [5]. There are 57 accepted human adenovirus types (HAdV-1 to 57) in 7 species (human adenovirus A to G). Among them, the most common pathogenic types are types 1-8, and types 3 and 7 are most prone to severe disease [6]. Of the cases with adenovirus pneumonia detected in the bronchoalveolar lavage fluid, 1 case was type 4, and the other cases were type 7. Adenovirus is mainly transmitted through air droplets. Infants, children, older people, and persons with low immune function are more likely to be infected and develop severe pneumonia [7]. Infants and young children are the main population, and the incidence is usually under 2 years old; the mean age of the patients was 1.7 years in this study.

The clinical manifestations of adenovirus pneumonia are related to age, serotype, and host immune function status. Studies have shown that age < 7 years, chronic underlying disease, posttransplantation, or immunocompromised function can increase the incidence of severe adenoviral pneumonia [8]. The incubation period after adenovirus infection is 3-8 days, and then, usually, high fever above 39°C appears in 1-2 days. The high fever lasts for 7-10 days, and the duration of severe cases can be longer. Compared with the nonsevere group, all children in the severe group had fever (100% vs. 65%, *P* < 0.001). Severe children have symptoms of dyspnea and hypoxia, lungs can be heard with a lot of wheezing and moist rales, the wheezing symptoms in the severe group were much greater than those in the nonsevere group (78% vs. 46%, *P* < 0.001), and the consolidation of the lungs (93% vs. 20%, *P* < 0.001) can be accompanied by pleural effusion (27% vs. 2%, *P* < 0.001), atelectasis (30% vs. 3%, *P* < 0.001), and even respiratory failure (100% vs. 0%, *P* < 0.001). Severe pneumonia cases often appear with mental depression, bloating, and diarrhea and are prone to multisystem complications such as toxic encephalopathy (9% vs. 0%, *P* < 0.001), myocardial damage (93% vs. 34%, *P* < 0.001), liver injury (86% vs. 22%, *P* < 0.001), and coagulation disorders (24% vs. 1%, *P* < 0.001). In this study, the rate of severe pneumonia children with two or more complications was as high as 68%. Therefore, early screening and treatment are particularly critical to improve the prognosis of children with severe adenoviral pneumonia.

In this paper, children with severe adenoviral pneumonia and the nonsevere group are analyzed in terms of biochemical indicators and coagulation function. Statistics showed that the levels of biochemical indicators such as AST (131.5 ± 50.8 vs. 51.9 ± 21.2, *P* < 0.001) and LDH-L (1423 ± 635 vs. 278 ± 195, *P* < 0.001) in children with severe adenoviral pneumonia were significantly higher than the nonsevere group. Children with severe pneumonia may suffer from both impaired pulmonary ventilation and pulmonary ventilation, which may lead to hypoxemia, severe microcirculation disturbances [9], insufficient tissue perfusion, and cell metabolism disorders. Meanwhile, pathogenic microorganisms and various inflammatory factors cause different degrees of damage to liver cells and cardiomyocytes, which increase cell membrane permeability and concentrations of AST and LDH in serum. Studies have found that the level of AST is related to the severity of adenovirus infection [10]. PCT is a functional protein and is a propeptide of calcitonin. When the body is severely infected, the PCT level can rise within 2-3 hours, the duration of the high level is 8-24 hours, and the half-life is 24-35 hours. The level of PCT in the body is positively correlated with the severity of inflammation [11]. The PCT level mainly reflects the severity of bacterial infection; pathogen test results showed that 66.30% (61/92) of the severe group had infection with other pathogens and 43.52% (84/193) for the nonsevere group. The incidence of bacterial infection was 25% (23/92) in the severe group and 11.92% (23/193) in the nonsevere group. The results of this study show that the PCT level (5.9 ± 2.7 vs. 0.9 ± 0.4, *P* < 0.001) of children with severe adenoviral pneumonia is significantly higher than that of the nonsevere group, which is considered for severe adenoviral pneumonia combined with multiple pathogenic infections (16.30% vs. 9.84%). D-dimer is a specific marker of the fibrinolytic system, which is an important indicator of secondary hyperfibrinolysis. In the state of severe pneumonia [12], the tissue will suffer from hypoxia, ischemia and acidosis, coupled with the direct invasion of pathogens and their toxins; those factors will cause vascular endothelial cell damage and collagen exposure, activation of the coagulation system, and the formation of a hypercoagulable state, thereby forming microthrombus. Increased D-dimer levels in plasma indicate secondary hyperfibrinolysis; thrombin generated during the coagulation process can activate the fibrinolytic system and reflect antifibrinolytic activity or concentration.

Although there are no obvious signs of embolism in children with severe pneumonia, D-dimer levels can reflect the body's coagulation status and assess the coagulation status of children. The incidence of coagulation disorders in the severe group was much higher than that in the nonsevere group (131.5 ± 50.8 vs. 51.9 ± 21.2, *P* < 0.001), and D-dimer levels in children with severe adenoviral pneumonia are significantly higher than those in the nonsevere group (6.04 ± 3.03 vs. 1.78 ± 0.99, *P* < 0.001). Therefore, the increase of D-dimer not only is helpful for understanding the body's coagulation and fibrinolysis status but also can be used as an index to reflect the severity and prognosis of pneumonia.

For children with pneumonia whose pathogen is not clear, if there is persistent fever, accompanied by wheezing, shortness of breath, and poor spirit, the antibiotic treatment is not effective. Laboratory tests suggest that PCT, LDH, AST, ferritin, D-dimer, and other indicators significantly increase, accompanied or not by multiple organ damage; in this case, we need to be vigilant against severe adenoviral pneumonia which should be paid sufficient attention and actively treated to improve the prognosis.

For adenoviral infections, there are no specific therapeutic drugs [13]. Studies on the treatment of human adenovirus infections by antiviral drugs such as cidofovir, ribavirin, ganciclovir, and interferon are limited, and there are reports that the application of cidofovir antiviral treatment for adenoviral pneumonia has achieved good results [14]. For patients with concomitant bacterial infections, especially children with severe adenoviral pneumonia, sensitive antibiotics are recommended for anti-infection [15]. In severe adenoviral infection, the virus activates the release of inflammatory mediators, which cause a systemic inflammatory response and even impairment of multiple organ functions [16]. Precise mechanisms of the immune-modulatory and anti-inflammatory effects of IVIG are unknown. Intravenous immunoglobulin can regulate the body's immunity, inhibit cytokine production, and neutralize viral antibodies and inflammatory factors; intravenous infusion of which can shorten the fever time and reduce the occurrence of complications [17, 18]. It has been reported that IVIG shows beneficial effects on pulmonary lesions in influenza pneumonia and mycoplasma pneumonia [19, 20]. In China, the National Health Commission of the People's Republic of China and the State Administration of Traditional Chinese Medicine organized and formulated the guideline for diagnosis and treatment of adenovirus pneumonia in children (2019 version) [21]; the recommended dosage of IVIG is 1 g/(kg·d) for 2 days. The use of glucocorticoids is a double-edged sword. In the clinic, the indications should be strictly controlled and carefully selected. The guideline [21] suggests that it can be used in the following situations: (i) Obvious symptoms of poisoning, encephalitis or encephalopathy, hemophagocytic syndrome and other complications;(ii) Sepsis; (iii) Continuous wheezing, the main imaging manifestations are bronchiolitis. It is recommended to choose intravenous methylprednisolone 1-2 mg/(kg·d) or the equivalent amount of hydrocortisone;, generally short-term use is appropriate. It can shorten the course of fever, suppress the body's excessive immune response, and improve the symptoms of poisoning. Adenoviral pneumonia showed necrotic changes; bronchial mucosal necrosis blocked the lumen and aggravated ventilation dysfunction. Therefore, bronchoalveolar lavage can clear sputum plugs and the airway. In this study, 76 cases (82%) of children with severe adenoviral pneumonia underwent bronchoalveolar lavage, and all had bronchial endometritis, and 7 cases had plastic bronchitis.

Children with severe adenoviral pneumonia have a high mortality rate and a long recovery period, and their mortality rate can be up to 10% [22]. Of the 285 cases, 2 children died (7.02%), one was severely infected with hemophagocytic syndrome and pulmonary hemorrhage, and the other was with ARDS. Severe adenoviral pneumonia is easily associated with impaired multiorgan function. Severe intrapulmonary lesions, ARDS, and severe coagulation abnormalities are mostly the direct causes of death in children [23], which is consistent with the 2 children who died in this study. Unfortunately, 14%-16% of the recovered patients may have sequelae of different degrees [24]; occlusive bronchitis and bronchiectasis are the most common complications in children with severe adenoviral pneumonia. Some severe adenoviral pneumonia cases may still hear wheezing in the lungs during recovery, accompanied by varying degrees of wheezing [25]. Therefore, treatment to improve small airway ventilation is still needed in the adenoviral pneumonia recovery period to improve the long-term prognosis of children as much as possible.

## 5. Conclusions

The conducted research clearly shows that children with adenoviral pneumonia were usually under 2 years old. Severe clinical manifestations and many complications are positively related to the severity of the disease. AST, LDH-L, PCT, ferritin, and D-dimer levels have important clinical implications for assessing disease severity. If the accurate assessment of the severity of the disease is evaluated sooner and if the treatment is performed in time, the child will be treated better.

## Figures and Tables

**Table 1:** Comparison of clinical symptoms in 285 children with adenovirus pneumonia.

Symptoms	Severe (*n* = 92)	Nonsevere (*n* = 193)	*χ* ^2^	*P*
Fever	92 (100%)	125 (65%)	40.655	*P* < 0.001
Cough	89 (97%)	185 (96%)	0.001	0.973
Wheezing	72 (78%)	86 (46%)	28.643	*P* < 0.001
Rales	80 (87%)	152 (79%)	2.767	0.096

**Table 2:** Comparison of complications in 285 children with adenoviral pneumonia.

Complication	Severe (*n* = 92)	Nonsevere (*n* = 193)	*χ* ^2^	*P*
Respiratory failure	92 (100%)	0 (0%)	280.444	*P* < 0.001
Pleural effusion	25 (27%)	3 (2%)	43.311	*P* < 0.001
Consolidation	86 (93%)	38 (20%)	138.015	*P* < 0.001
Atelectasis	28 (30%)	6 (3%)	44.278	*P* < 0.001
Liver injury	79 (86%)	42 (22%)	104.805	*P* < 0.001
Myocardial damage	86 (93%)	65 (34%)	89.434	*P* < 0.001
Electrolyte disturbances	56 (60%)	22 (11%)	76.704	*P* < 0.001
Diarrhea	16 (17%)	28 (15%)	0.397	0.529
Coagulation disorders	22 (24%)	2 (1%)	39.365	*P* < 0.001
Toxic encephalopathy	8 (9%)	0 (0%)	14.227	*P* < 0.001
≥2	68 (74%)	16 (8%)	129.070	*P* < 0.001

**Table 3:** Comparison of peripheral blood tests in 285 children with adenovirus pneumonia.

	Severe	Nonsevere	*T*	*P*
WBC (∗10^9^/l)	7.5 ± 3.3	7.7 ± 2.3	0.272	0.787
CRP (mg/l)	38.3 ± 19.4	27.0 ± 13.3	1.988	0.053
PCT (ng/ml)	5.9 ± 2.7	0.9 ± 0.4	9.878	*P* < 0.001
SAA	144.7 ± 54.2	13.0 ± 66.7	0.817	0.418
ESR (mm/h)	38.4 ± 15.7	32.0 ± 18.9	0.801	0.428
Ferritin (ng/ml)	1 945 ± 1 001	465 ± 195	9.136	*P* < 0.001
AST (U/l)	131.5 ± 50.8	51.9 ± 21.2	8.255	*P* < 0.001
LDH-L (U/l)	1 423 ± 635	278 ± 195	10.819	*P* < 0.001
D-dimer (mg/l)	6.04 ± 3.03	1.78 ± 0.99	7.706	*P* < 0.001

## Data Availability

All data generated or analyzed during this study are included in this article. All data and materials are presented in Material and Methods and Results as shown in figures and tables.

## References

[1] Berciaud S., Rayne F., Kassab S. (2012). Adenovirus infections in Bordeaux University Hospital 2008-2010: clinical and virological features. *Journal of Clinical Virology*.

[2] Kajon A., Lynch J. (2016). Adenovirus: epidemiology, global spread of novel serotypes, and advances in treatment and prevention. *Seminars in Respiratory and Critical Care Medicine*.

[3] Xie L., Zhang B., Zhou J. (2018). Human adenovirus load in respiratory tract secretions are predictors for disease severity in children with human adenovirus pneumonia. *Virology Journal*.

[4] Harris J. A., Murphy J. A. (2011). Retigabine (ezogabine) as add-on therapy for partial-onset seizures: an update for clinicians. *Thorax*.

[5] Xie L., Zhang B., Xiao N. (2019). Epidemiology of human adenovirus infection in children hospitalized with lower respiratory tract infections in Hunan, China. *Journal of Medical Virology*.

[6] Li Y., Zhou W., Zhao Y. (2015). Molecular typing and epidemiology profiles of human adenovirus infection among paediatric patients with severe acute respiratory infection in China. *PLoS One*.

[7] Pfortmueller C. A., Barbani M. T., Schefold J. C., Hage E., Heim A., Zimmerli S. (2019). Severe acute respiratory distress syndrome (ARDS) induced by human adenovirus B21: report on 2 cases and literature review. *Journal of Critical Care*.

[8] Gray G. C., McCarthy T., Lebeck M. G. (2007). Genotype prevalence and risk factors for severe clinical adenovirus infection, United States 2004-2006. *Clinical Infectious Diseases*.

[9] Giannitti F., Diab S. S., Uzal F. A. (2012). Infection with a Hepatozoon sp. closely related to Hepatozoon felis in a wild Pampas gray fox (Lycalopex -Pseudalopex -gymnocercus) co-infected with canine distemper virus. *Veterinary Parasitology*.

[10] Hwang S. M., Park D. E., Yang Y. I. (2013). Outbreak of febrile respiratory illness caused by adenovirus at a South Korean military training facility: clinical and radiological characteristics of adenovirus pneumonia. *Japanese Journal of Infectious Diseases*.

[11] Krüger S., Ewig S., Kunde J. (2010). Assessment of inflammatory markers in patients with community-acquired pneumonia — influence of antimicrobial pre-treatment. *Clinica Chimica Acta*.

[12] Di Ge L. H., Yang F., Zhao N., Yang Y., Zhang H., Chen Y. (2019). Development of hydroxamate-based histone deacetylase inhibitors of bis-substituted aromatic amides with antitumor activities. *MedChemComm*.

[13] Vasileiou S., Turney A. M., Kuvalekar M. (2020). Rapid generation of multivirus-specific T lymphocytes for the prevention and treatment of respiratory viral infections. *Haematologica*.

[14] Kim S. J., Kim K., Park S. B., Hong D. J., Jhun B. W. (2015). Outcomes of early administration of cidofovir in non-immunocompromised patients with severe adenovirus pneumonia. *PLoS One*.

[15] Huang M., Luo R., Fu Z. (2017). Risk factors for poor prognosis in children with severe adenovirus pneumonia. *Chinese Journal of Contemporary Pediatrics*.

[16] Fu Y., Tang Z., Ye Z. (2019). Human adenovirus type 7 infection causes a more severe disease than type 3. *BMC Infectious Diseases*.

[17] Chaigne B., Mouthon L. (2017). Mechanisms of action of intravenous immunoglobulin. *Transfusion and Apheresis Science*.

[18] De Ranieri D. (2017). Intravenous immunoglobulin and its clinical applications. *Pediatric Annals*.

[19] Rhim J. W., Lee K. Y., Youn Y. S., Kang J. H., Kim J. C. (2011). Epidemiological and clinical characteristics of childhood pandemic 2009 H1N1 virus infection: an observational cohort study. *BMC Infectious Diseases*.

[20] Youn Y. S., Lee S. C., Rhim J. W., Shin M. S., Kang J. H., Lee K. Y. (2014). Early additional immune-modulators for Mycoplasma pneumoniae pneumonia in children: an observation study. *Infect. Chemother*.

[21] National Health Commission of the People’s Republic of China, the State Administration of Traditional Chinese Medicine (2019). Guideline for diagnosis and treatment of adenovirus pneumonia in children (2019 version). *Chinese Journal of Clinical Infectious Diseases*.

[22] Hong J.-Y., Lee H.-J., Piedra P. A. (2001). Lower respiratory tract infections due to adenovirus in hospitalized Korean children: epidemiology, clinical features, and prognosis. *Clinical Infectious Diseases*.

[23] Yu Z., Zeng Z., Zhang J. (2016). Fatal community-acquired pneumonia in children caused by re-emergent human adenovirus 7d associated with higher severity of illness and fatality rate. *Scientific Reports*.

[24] Berkley J. A., Munywoki P., Ngama M. (2010). Viral etiology of severe pneumonia among Kenyan infants and children. *JAMA*.

[25] Ions R., Narayanan M., Browning M., Gaillard E. A., Stiefel G., Tang J. W. (2018). Case presentation: persistent adenovirus B3 infections associated with bronchiolitis obliterans treated with cidofovir in a child with mosaic tetrasomy 9p. *BMC Infectious Diseases*.

